# Decreased expression of autophagy‐related genes in the complete remission phase of acute myeloid leukemia

**DOI:** 10.1002/mgg3.1872

**Published:** 2022-02-06

**Authors:** Parisa Tandel, Reza Ranjbaran, Eqbal Ebrahimi, Alireza Rezvani, Mani Ramzi, Gholamhossein Tamaddon

**Affiliations:** ^1^ Diagnostic Laboratory Sciences and Technology Research Center, School of Paramedical Sciences Shiraz University of Medical Sciences Shiraz Iran; ^2^ Hematology and Oncology Department, School of Medicine Shiraz University of Medical Sciences Shiraz Iran; ^3^ Hematology Research Center Shiraz University of Medical Sciences Shiraz Iran

**Keywords:** acute myeloid leukemia, autophagy, follow‐up, remission

## Abstract

**Background:**

Autophagy is a conserved recycling process in cells. However, the effects of autophagy on the remission and treatment response of acute myeloid leukemia (AML) patients have not been clarified.

**Methods:**

The expression of *MAP1LC3B*, *ATG5*, *ATG10*, *RB1CC1*, and *AMBRA1* genes was assessed in 32 newly diagnosed AML patients, 18 complete remission (CR) patients, and seven relapsed patients, as well as 15 controls, by real‐time polymerase chain reaction (PCR).

**Results:**

The expression of all five genes was significantly higher in the newly diagnosed AML patients as compared to the controls (*p* < 0.0001). The *MAP1LC3B*, *ATG5*, *ATG10*, *RB1CC1,* and *AMBRA1* gene expression significantly reduced in CR patients compared to newly diagnosed AML patients (*p* = 0.006, 0.003, 0.0002, 0.006, and 0.004, respectively). The *AMBRA1* gene expression was significantly higher in the relapsed cases as compared to both newly diagnosed (*p* = 0.01) and CR patients (*p* = 0.03). Moreover, a significant positive correlation was observed between the expression of *MAP1LC3B* (*r* = 0.739, *p* = 0.000001), *ATG5* (*r* = 0.682, *p* = 0.00001), and *ATG10* (*r* = 0.586, *p* = 0.0004) genes and white blood cell (WBC) count in patients at diagnosis.

**Conclusion:**

The expression of *MAP1LC3B*, *ATG5*, *ATG10*, *RB1CC1,* and *AMBRA1* genes can be examined to follow‐up the remission of AML and the patient's response to treatment.

## Graphical abstract

Autophagy is known as an effective pathway in biological processes, but their unknowns are still secret in leukemia and response to treatment. The autophagy‐related genes might be changed and useful to remission follow‐up. In this study, we try to introduce the impact of autophagy‐related genes in pathogenesis, treatment response, and remission of acute myeloid leukemia.

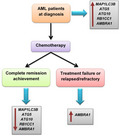

## 1 INTRODUCTION

Autophagy is a conserved recycling system in cells, which leads to the degradation of damaged cytoplasmic components by lysosomes (Parzych & Klionsky, 2014). It is an important pathway to maintain cellular hemostasis; its activation helps the cells survive when exposed to stressors (Chun & Kim, 2018). This process plays a dual role in cancer initiation and progression. Evidence shows that decreased autophagy can lead to accelerated tumorigenesis. On the other hand, autophagy induction can increase the proliferation and survival of tumor cells under adverse conditions (Chen & Debnath, 2010); therefore, it remains a controversial issue in tumorigenesis and cancer.

Disruption of autophagy may play an important role in the initiation and development of leukemias, including acute myeloid leukemia (AML) (Auberger & Puissant, 2017). AML is an aggressive hematological malignancy, which results from the accumulation of abnormal myeloid progenitors in the bone marrow and peripheral blood (Saultz & Garzon, 2016). The primary treatment for AML patients is chemotherapy (Tamamyan et al., 2017), and the main goal of treatment is to induce complete remission (CR) and prevent relapse (Dohner et al., 2010).

Despite attaining CR in most patients, the relapse rate remains high after treatment (Bryan & Jabbour, 2015). Recent studies have revealed that drug resistance might be one of the most significant causes of treatment failure in AML patients (Shaffer et al., 2012; Zhang et al., 2019). Aberrant activation of several signaling pathways, such as autophagy, contributes to the drug resistance mechanisms of AML (Chen et al., 2016; Zhang et al., 2019). Autophagy enhancement can play a role as a chemo‐resistance mechanism during chemotherapy of AML patients (Evangelisti et al., 2015; Piya & Kornblau, 2016; Piya et al., 2017).

According to recent studies, autophagy inhibition via suppression of autophagy‐related genes or using autophagy inhibitory drugs can enhance the cytotoxicity of chemotherapy drugs in AML cell lines (Nourkeyhani et al., 2016; Palmeira‐Dos‐Santos et al., 2014; Piya & Kornblau, 2016). However, the effects of autophagy on response to therapy and remission in AML patients have not been clarified, and further investigation is necessary. The present study aimed to evaluate the expression of *MAP1LC3B* (OMIM accession number: * 609604), *ATG5* (OMIM accession number: * 604261), *ATG10* (OMIM accession number: * 610800), *RB1CC1* (OMIM accession number: * 606837), and *AMBRA1* (OMIM accession number: * 611359) genes as the main genes in the autophagy pathway at diagnosis and in the CR and relapse phases of AML.

## 2 MATERIALS AND METHODS

### 2.1 Samples and patients

In this case–control study, the whole blood samples were collected from 32 newly diagnosed AML patients (new cases), who did not have a history of chemotherapy. These patients were classified according to the treatment regimen (AML‐M3 and non‐M3 AML) at the Hematology Department of Namazi Hospital, affiliated to Shiraz University of Medical Sciences, Shiraz, Iran.

The patients received the standard‐of‐care regimens (cytarabine + anthracycline for non‐M3 AML patients and all‐trans retinoic acid [ATRA] + arsenic trioxide for AML‐M3 patients), and after the induction phase, 18 patients attained CR. Patients who expired were excluded from the study. We also included seven AML patients in the relapse phase. Moreover, we recruited 15 healthy individuals without cancer or hematological diseases as the control group; they were matched with AML patients in terms of age and gender.

### 2.2 RNA extraction and cDNA synthesis

Total RNA was extracted from whole blood samples using TRIzol (Invitrogen, Carlsbad, CA, USA), according to the manufacturer's instructions. Subsequently, the extracted RNA concentrations were measured by a NanoDrop instrument (Hellma, Denmark). Next, 0.4 μg of each RNA was reverse‐transcribed into cDNA to a final volume of 10 μl, according to the instructions of the PrimeScript First‐Strand cDNA Synthesis Kit (Takara, Shiga, Japan).

### 2.3 Quantitative real‐time polymerase chain reaction (qRT‐PCR)

AlleleID 7.0 and Gene Runner were used to design the sequences of specific primers. A qRT‐PCR was performed to evaluate the expression of *MAP1LC3B* (GenBank Ref Seq no: NM_022818.5), *ATG5* (GenBank Ref Seq no: NM_004849.4), *ATG10* (GenBank Ref Seq no: NM_031482.5), *RB1CC1* (GenBank Ref Seq no: NM_014781.5), and *AMBRA1* (GenBank Ref Seq no: NM_001267782.2) genes, using Qiagen real‐time PCR cycler (Rotor Gene, Germany). Beta‐actin was used as an endogenous control for normalization of mRNA expression between different samples. For each RT‐PCR reaction, 1 μl of cDNA, 10 μl of SYBR Green PCR Master Mix (SYBR Premix Ex Taq™II, Tli RNaseH Plus Yektatajhiz, Iran), 8.2 μl of nuclease‐free water, 0.4 μl of forward primer, and 0.4 μl of reverse primer in a final volume of 20 μl were used. The fold changes in *MAP1LC3B*, *ATG5*, *ATG10*, *RB1CC1*, and *AMBRA1* gene expression were analyzed using the 2^−∆∆CT^ method.

### 2.4 Statistical analysis

Statistical analysis was performed using GraphPad Prism 8.0.2 (Graph Pad Software Inc., San Diego, California, USA) and SPSS version 25.0 (SPSS IBM, Chicago, IL, USA). Mann–Whitney test, Wilcoxon test, paired *t*‐test, and unpaired *t*‐test were performed to compare the gene expression between each two groups. Fold changes were calculated using the 2^−∆∆CT^ method. Moreover, the correlations between gene expression and the clinical characteristics of patients were analyzed using Pearson's correlation coefficient (*r*) test. *p*‐value <0.05 was considered to be statistically significant in all tests.

## 3 RESULTS

### 3.1 Patients' clinical characteristics

The clinical characteristics of 32 newly diagnosed AML patients, 18 AML patients in the CR phase, and seven patients in the relapse phase, as well as 15 healthy controls are presented in Table 1. Cytogenetic status of AML pateints at diagnosis and in the CR and relapse phases showed in Table 2.

**TABLE 1:** The clinical information of AML patients at diagnosis and in the complete remission and relapse phases and the healthy controls

	New case	Complete remission	Relapse	Control
Sex, male/female	16/16	8/10	4/3	9/6
Age, median (range)	52 (24–75)	50 (24–75)	50 (24–78)	44 (27–73)
WBC (*10^9^/L), median (range)	32.8 (0.5–166)	2.6 (0.4–16.9)	5.9 (0.9–15)	6.7 (5.2–10)
PLT(*10^9^/L), median (range)	65.8 (6–227)	87.2 (8–230)	70 (4–200)	252 (196–321)
Hb (g/L), median (range)	7.9 (4.4–11.2)	8.3 (7.2–10.4)	7.9 (5.1–10.3)	14.6 (13.2–15.8)
BM blast %, median (range)	73 (40–90)	<5 (1–3)	57 (30–77)	–
Classification (AML M3/non‐M3)	5/27	3/15	1/6	–

Abbreviations: BM, bone marrow; Hb, hemoglobin; PLT, platelet; WBC, white blood cells.

**TABLE 2:** Cytogenetic status of AML pateints at diagnosis and in the complete remission and relapse phases

New case *n* = 32		Complete remission *n* = 18		Relapse *n* = 7	
AML‐NOS	16	AML‐NOS	10	AML‐NOS	6
*t*(15;17) (q24;q21); PML‐RARA	5	*t*(15;17) (q24;q21); PML‐RARA	3	*t*(15;17) (q24;q21); PML‐RARA	1
*t*(8;21) (q22;q22.1);RUNX1‐RUNX1T1	7	*t*(8;21) (q22;q22.1);RUNX1‐RUNX1T1	4		
*t*(16;16) (p13.1;q22);CBFB‐MYH11	4	*t*(16;16) (p13.1;q22);CBFB‐MYH11	1		

Abbreviation: AML‐NOS, acute myeloid leukemia‐not otherwise specified.

### 3.2 Expression of *MAP1LC3B* , *ATG5* , *ATG10* , *RB1CC1,* and *AMBRA1* genes in newly diagnosed AML patients and control groups

**FIGURE 1:**
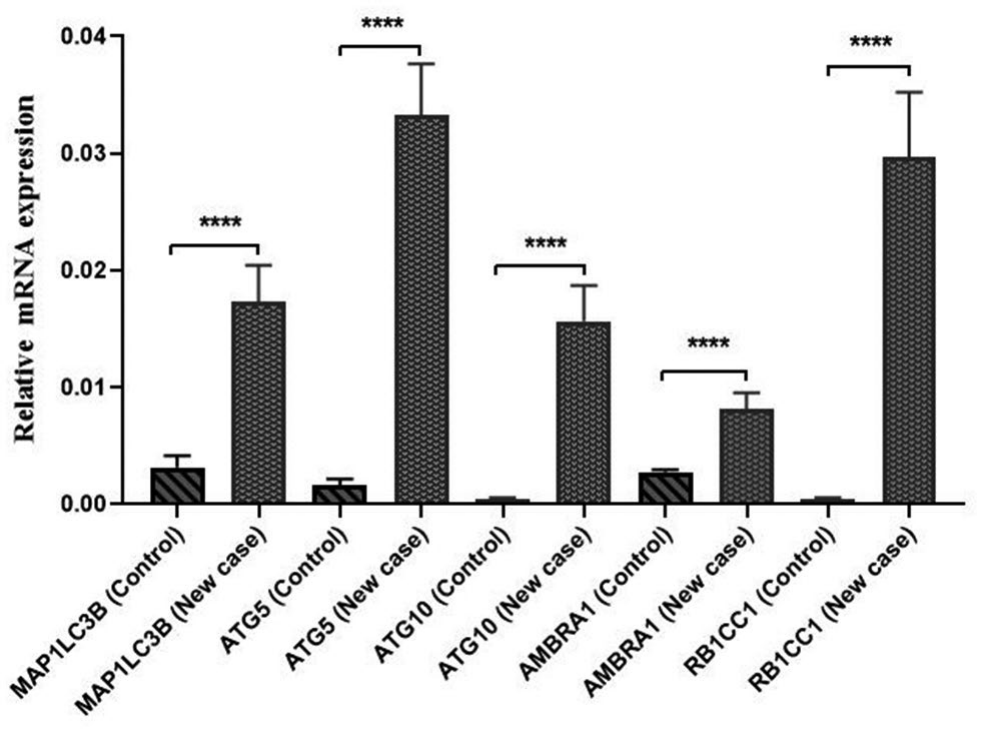
Comparison of the expression of *MAP1LC3B*, *ATG5*, *ATG10*, *RB1CC1*, and *AMBRA1* genes between newly diagnosed AML patients and controls. The expression of these genes significantly increased in AML patients versus the controls ^(^****p < 0.0001)

The mRNA expression of *MAP1LC3B*, *ATG5*, *ATG10*, *RB1CC1*, and *AMBRA1* genes was evaluated in 32 newly diagnosed AML (new case) patients and 15 healthy controls. The results showed that the expression of *MAP1LC3B*, *ATG5*, *ATG10*, *RB1CC1*, and *AMBRA1* genes was significantly upregulated in AML patients as compared to the control group (7.18, 27.6, 36.8, 69.7, and 3.6 folds, respectively) (*p* < 0.0001). The details of these results are shown in Figure 1.

### 3.3 Changes in the expression of *MAP1LC3B* , *ATG5* , *ATG10* , *RB1CC1,* and *AMBRA1* genes in AML patients in the different disease phases

**FIGURE 2:**
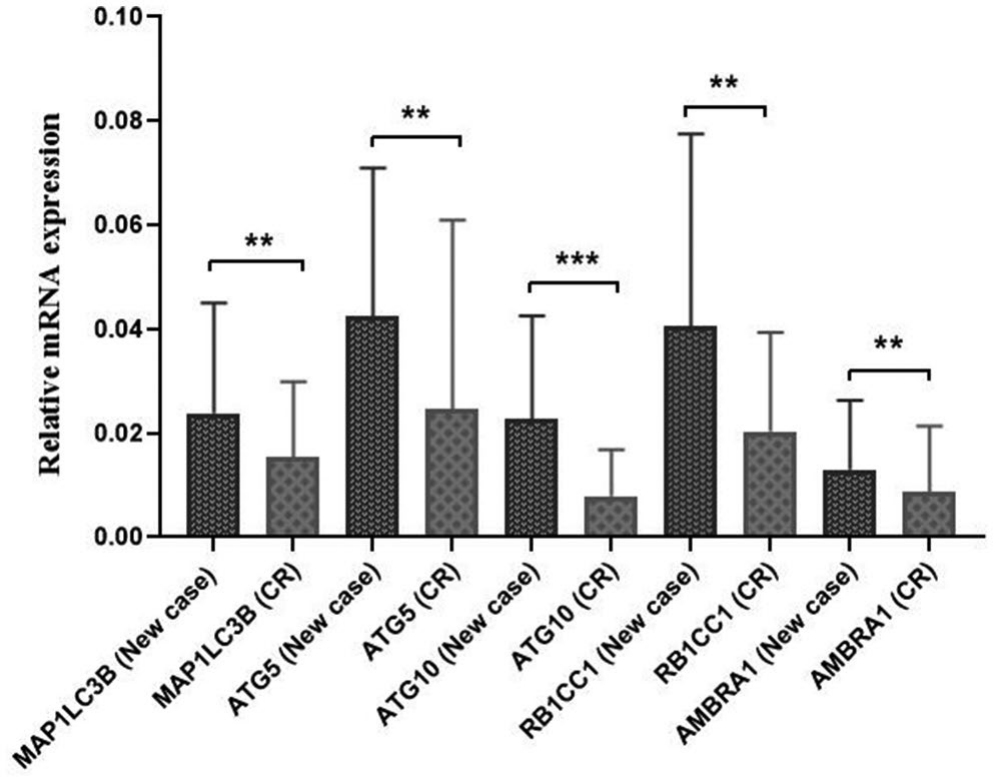
The relative expression of *MAP1LC3B*, *ATG5*, *ATG10*, *RB1CC1*, and *AMBRA1* genes in CR patients versus newly diagnosed AML patients. All genes showed significantly lower expression levels in AML‐CR patients compared to newly diagnosed AML cases (**p < 0.01, ***p < 0.001)

The expression of *MAP1LC3B*, *ATG5*, *ATG10*, *RB1CC1*, and *AMBRA1* genes was investigated in 18 newly diagnosed AML patients and 18 AML‐CR patients in the same cases. The results showed that the expression of *MAP1LC3B*, *ATG5*, *ATG10*, *RB1CC1*, and *AMBRA1* genes was significantly downregulated in CR patients as compared to newly diagnosed AML patients (0.71, 0.73, 0.5, 0.72, and 0.75 folds, respectively) (*p* = 0.006, 0.003, 0.0002, 0.006, and 0.004, respectively). The relative expression of these genes in CR patients compared to newly diagnosed AML patients is shown in Figure 2.

**FIGURE 3:**
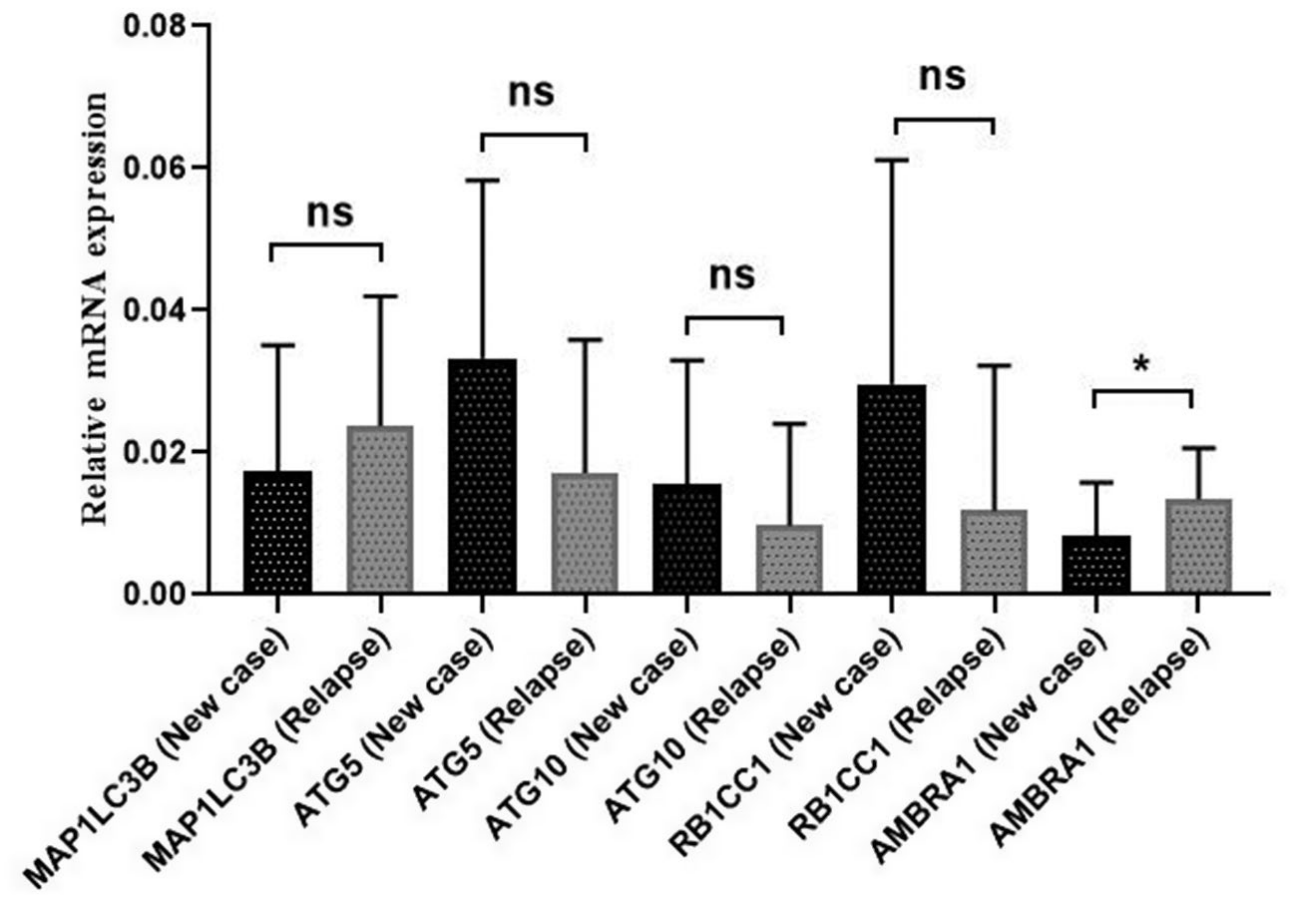
Comparison of the expression of *MAP1LC3B*, *ATG5*, *ATG10*, *RB1CC1*, and *AMBRA1* genes between relapsed and newly diagnosed AML patients. The *AMBRA1* gene expression was significantly higher in relapsed patients as compared to newly diagnosed cases (*p < 0.05)

Moreover, the expression of *MAP1LC3B*, *ATG5*, *ATG10*, *RB1CC1*, and *AMBRA1* genes was assessed in seven AML patients in the relapse phase and compared with that of 32 newly diagnosed AML patients. There was no significant difference in the expression of *MAP1LC3B, ATG5, ATG10*, and *RB1CC1* genes between relapsed and newly diagnosed cases (*p* > 0.05). The *AMBRA1* gene expression significantly increased in patients with relapse as compared to newly diagnosed AML patients (2.1 folds) (*p* = 0.01); the details are presented in Figure 3.

**FIGURE 4:**
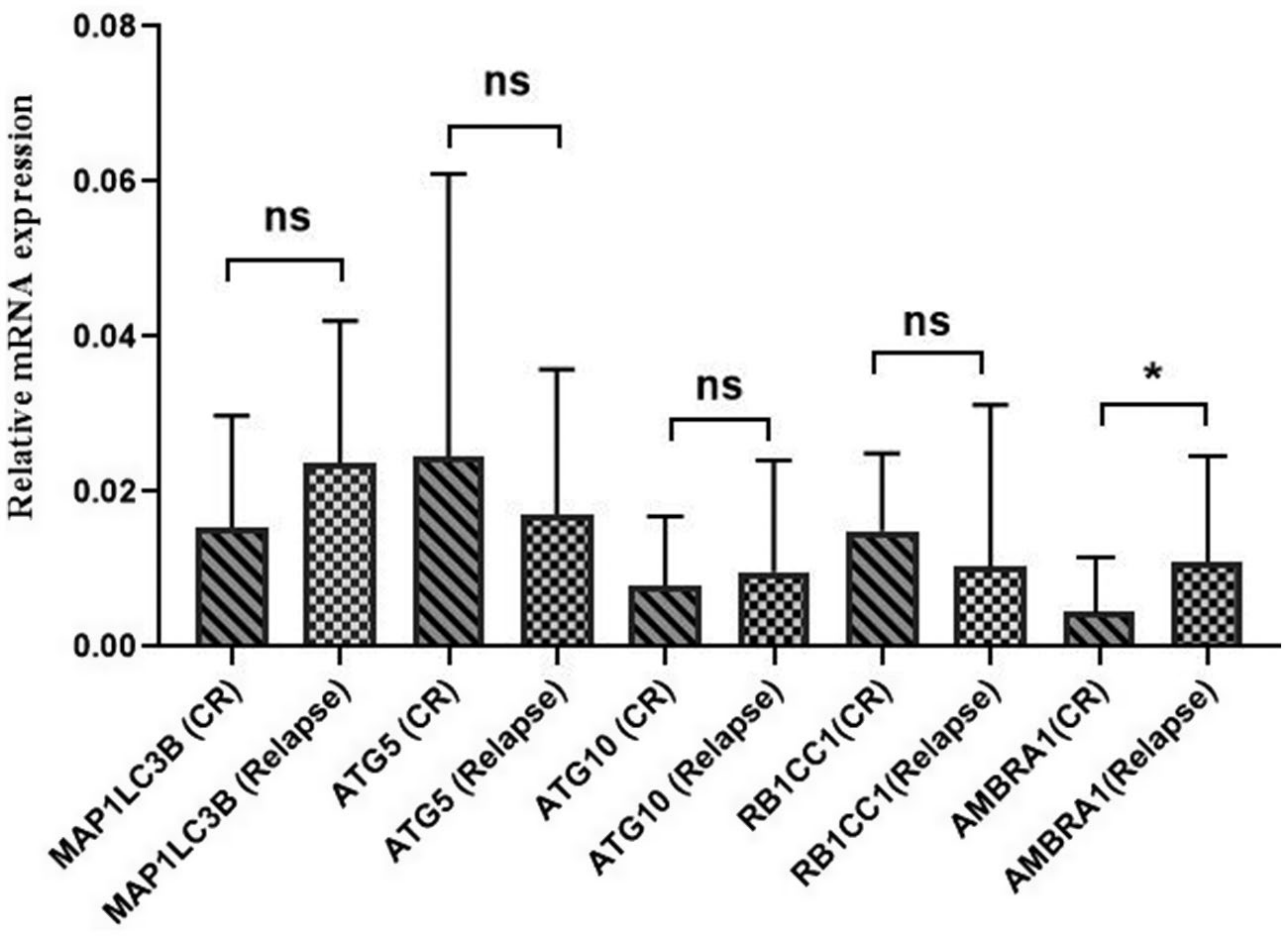
The expression of *MAP1LC3B*, *ATG5*, *ATG10*, *RB1CC1*, and *AMBRA1* genes in relapsed AML patients as compared to AML‐CR patients. The expression of *AMBRA1* gene was significantly higher in relapsed patients as compared to CR patients (*p < 0.05)

Moreover, to determine the role of autophagy‐related genes in the chemo‐resistance of AML patients, we compared the expression of *MAP1LC3B*, *ATG5*, *ATG10*, *RB1CC1*, and *AMBRA1* genes in seven relapsed cases versus 18 CR cases. The results showed that the expression of *MAP1LC3B*, *ATG5*, *ATG10*, and *RB1CC1* genes was not significantly different between the relapse and CR groups (*p* > 0.05). However, the expression of *AMBRA1* gene was significantly higher in relapsed patients as compared to CR patients (2.8‐folds) (*p* = 0.03); this comparison is presented in Figure 4.

### 3.4 Correlation of *MAP1LC3B* , *ATG5* , *ATG10* , *RB1CC1* , and *AMBRA1* gene expression with the patients' clinical characteristics

The correlations between the expression of *MAP1LC3B*, *ATG5*, *ATG10*, *RB1CC1*, and *AMBRA1* genes and age, gender, hemoglobin (Hb) level, white blood cell (WBC) count, platelet count, bone marrow blast percentage, and subtype at diagnosis and in the CR and relapse phases were investigated. There was no significant association between the expression of these genes and age, gender, Hb level, platelet count, bone marrow percentage, and subtype of patients at diagnosis.

However, a significant positive correlation was found between the expression of *MAP1LC3B* (*r* = 0.739, *p* = 0.000001), *ATG5* (*r* = 0.682, *p* = 0.00001), and *ATG10* (*r* = 0.586, *p* = 0.0004) genes and WBC count. Therefore, upregulation of *MAP1LC3B*, *ATG5*, and *ATG10* genes was associated with a high WBC count at diagnosis. In CR patients, no significant correlation was observed between the expression of *MAP1LC3B*, *ATG5*, *ATG10*, *RB1CC1*, and *AMBRA1* genes and the patients' clinical characteristics (*p* > 0.05). Also, the results did not indicate a significant correlation between the expression of these genes and the clinical characteristics of relapsed patients (*p* > 0.05).

### 3.5 Correlation between the expression of *MAP1LC3B* , *ATG5* , *ATG10* , *RB1CC1* , and *AMBRA1* genes and CD34+ marker

**FIGURE 5:**
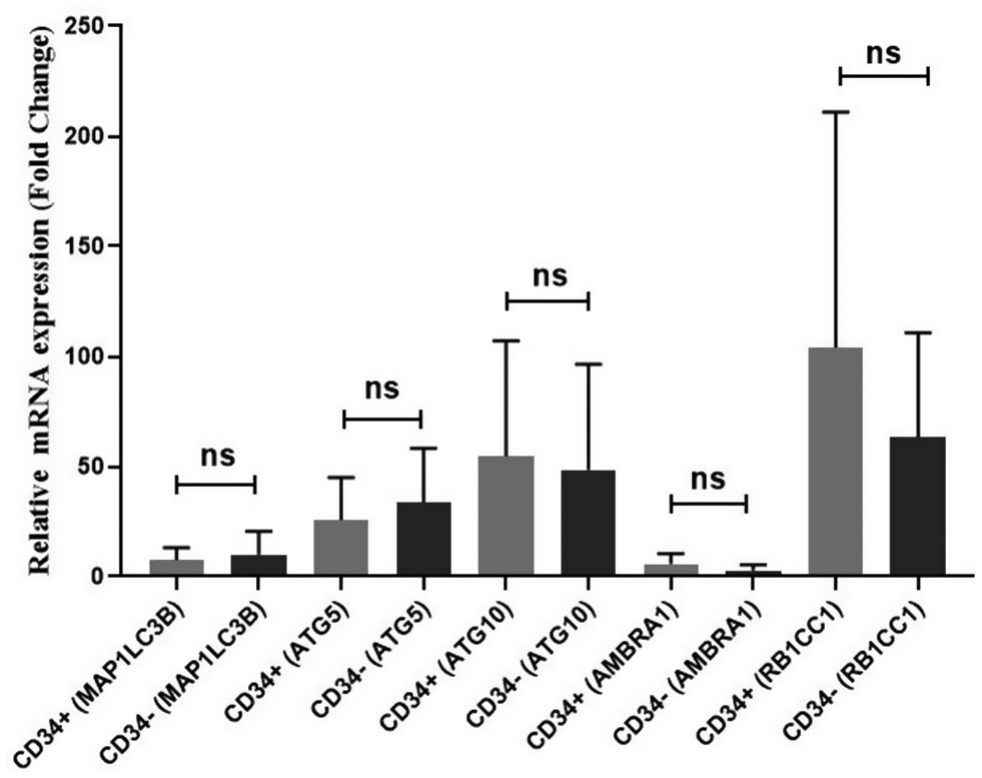
Correlation of *MAP1LC3B*, *ATG5*, *ATG10*, *RB1CC1*, and *AMBRA1* gene expression with CD34 marker. There was no significant association between the expression of these genes and CD34 marker (ns, not significant)

The relationship between the expression of *MAP1LC3B*, *ATG5*, *ATG10*, *RB1CC1*, and *AMBRA1* genes and CD34+ marker was examined in this study. According to the results, there was no significant correlation between the expression of *MAP1LC3B*, *ATG5*, *ATG10*, *RB1CC1*, and *AMBRA1* genes and CD34+ marker (*p* > 0.05); the results are presented in Figure 5.

### 3.6 Correlations between autophagy‐related genes

A statistical analysis was performed to evaluate the correlations between *MAP1LC3B*, *ATG5*, *ATG10*, *RB1CC1*, and *AMBRA1* genes. The results showed a significant positive correlation between *MAP1LC3B*, *ATG5*, *ATG10*, and *RB1CC1* genes (*p* < 0.05). Besides, the expression of *AMBRA1* gene had a significant positive correlation with *ATG5* and *RB1CC1* genes (*p* < 0.05).

## 4 DISCUSSION

Autophagy is a conserved, self‐degradative, multistep process, which plays a critical role in maintaining cellular hemostasis. This pathway consists of several phases, including induction, autophagosome nucleation, elongation and maturation, and lysosomal fusion and degradation. Various proteins contribute to the autophagy process, called autophagy‐related proteins (ATG). FIP200 is an essential protein for autophagy induction, encoded by the *RB1CC1* gene. This protein, along with ULK1, ATG13, and ATG101, forms the ULK complex, which activates autophagy under various conditions, such as starvation and treatment with rapamycin. Autophagosome nucleation is triggered by activation of class III phosphatidylinositol 3‐kinase (PI3KC3) complex. PIK3C3, Beclin1, AMBRA1, and P150 form the core of the PI3KC3 complex. Generally, the vesicle elongation and maturation phase depends on two ubiquitin‐like conjugation reactions: ATG5‐ATG12 and LC3‐PE conjugation. ATG7 (an E1‐like enzyme), as well as ATG10 and ATG3 (E2‐like enzymes), is involved in these conjugation reactions. During the vesicle elongation and maturation phase, ATG4 cleaves LC3 to LC3I. Then, LC3I is converted into LC3II, which remains on the autophagosomes membrane and is used as an autophagy marker (Badadani, 2012; Dikic & Elazar, 2018; Glick et al., 2010).

Autophagy may affect the initiation and progression of leukemias. Leukemic cells can utilize autophagy to provide their metabolic needs for proliferation and survival. Also, autophagy activation can protect leukemic cells against oxidative stress; therefore, it can promote leukemogenesis, as well as drug resistance following treatment (Mourgues et al., 2015; Piya & Kornblau, 2016; Rothe et al., 2014).

The standard initial treatment for AML patients consists of cytarabine in combination with an anthracycline for non‐M3 AML and ATRA in combination with arsenic trioxide for M3‐AML. Despite achieving CR in the majority of patients, the rate of recurrence remains high following treatment (Tamamyan et al., 2017). According to emerging data, drug resistance is known as one of the most important causes of treatment failure in AML patients (Zhang et al., 2019). Recent studies have shown that activation of autophagy upon chemotherapy can be involved in the drug resistance of AML patients (Chen et al., 2016; Niu et al., 2019; Rothe et al., 2019).

In the present study, the expression of key autophagy‐related genes, including *MAP1LC3B*, *ATG5*, *ATG10*, *RB1CC1*, and *AMBRA1*, was evaluated at diagnosis and in the CR and relapse phases of AML patients. The results showed that the expression of *MAP1LC3B*, *ATG5*, *ATG10*, *RB1CC1*, and *AMBRA1* genes significantly increased by 7.18, 27.6, 36.8, 69.7, and 3.6‐folds, respectively in AML patients as compared to the healthy controls (*p* < 0.0001) (Figure 1). This finding is in line with the results reported by Hu XY et al. on acute leukemia patients, which suggested that autophagy activation and expression of *BECN1* and *MAP1LC3B* genes were significantly higher in de novo patients as compared to the controls (Hu et al., 2011). Our results showed that the upregulation of autophagy genes might be related to the pathogenesis of AML.

Additionally, Zare Abdollahi et al. reported that the expression of *BECN1* gene was significantly lower in AML patients with intermediate and unfavorable cytogenetic risks as compared to the normal controls. However, in the favorable subtypes of AML, the *BECN1* gene expression did not show any significant changes (Zare‐Abdollahi et al., 2016). Yun Lian et al. also found no significant differences in the expression of *BECN1* and *ATG5* genes between AML patients and controls (Lian et al., 2018). This discrepancy between the results might be due to the dual role of autophagy in leukemogenesis, different AML subtypes, or the genetic background of the studied populations.

In this study, the expression level of *AMBRA1* gene was significantly higher in the relapsed cases as compared to both diagnosis and CR cases (2.1 and 2.8‐folds, respectively; *p* = 0.01 and 0.03, respectively) (Figures 3 and 4); this might be due to the possible role of *AMBRA1* gene in the chemo‐resistance of relapsed/refractory AML patients. Moreover, the expression of *MAP1LC3B* gene, as an important marker of autophagy, increased in patients with relapse compared to newly diagnosed and CR cases; however, it was not statistically significant (Figures 3 and 4). Therefore, further research is required on a larger group of relapsed patients to determine the exact role of *MAP1LC3B* in relapsed/refractory AML.

Moreover, our results demonstrated that in the CR phase, the expression level of *MAP1LC3B, ATG5, ATG10, RB1CC1*, and *AMBRA1* genes was significantly reduced (0.71, 0.73, 0.5, 0.72, and 0.75‐folds, respectively) as compared to newly diagnosed AML patients (*p* = 0.006, 0.003, 0.0002, 0.006, and 0.004, respectively) (Figure 2). This finding is in line with our previous study, which revealed that the expression of *BECN1* gene in AML‐CR patients was significantly lower than newly diagnosed cases (Tandel et al., 2020). This result shows that decreased autophagy activity might be beneficial in achieving CR in patients. Therefore, targeting autophagy‐related genes might help to ameliorate the treatment outcomes of AML patients.

Additionally, the expression of *MAP1LC3B, ATG5, ATG10, RB1CC1*, and *AMBRA1* genes may be used as a therapeutic biomarker to follow‐up the CR status. In this regard, Palmeira dos Santos et al. revealed that autophagy blockade by 3‐methyladenine (3MA) could enhance cytarabine cytotoxicity against the HL‐60 cell line (Palmeira‐Dos‐Santos et al., 2014). Piya et al. revealed that ATG7 plays a significant role in the AML cell chemo‐resistance. In other words, the suppression of ATG7 increases the sensitivity of these cells to cytarabine‐induced cell death (Piya & Kornblau, 2016). Lian et al. reported that the upregulation of *ATG5* and *BECN1* genes could be associated with a poor prognosis, and downregulation of these genes could be related to a high CR rate in AML patients (Lian et al., 2018).

The results of this study also revealed that the overexpression of *MAP1LC3B* (*r* = 0.739, *p* = 0.000001), *ATG5* (*r* = 0.682, *p* = 0.00001), and *ATG10* (*r* = 0.586, *p* = 0.0004) genes was related to a high WBC count at diagnosis. Based on our results and studies on the role of autophagy in myeloid cell proliferation, differentiation, and apoptosis (Mourgues et al., 2015; Palmeira‐Dos‐Santos et al., 2014; Rothe et al., 2014), an increase in autophagy activity may affect the myeloid cell functions in AML patients; however, further studies are needed to confirm this finding.

The expression of CD34+ marker is associated with multi‐drug resistance (MDR) in AML and myelodysplastic syndrome (MDS) patients (List, 1996; Sonneveld et al., 1993). Several studies have shown that the increased number of CD34+ blasts could be related to relapse and disease progression in both AML and MDS (Macedo et al., 1996; Suarez et al., 2004). Moreover, CD34+ blasts show more resistance to chemotherapy and apoptosis compared to normal CD34+ fractions (Konopleva et al., 2002; Suarez et al., 2004). Considering the role of CD34+ marker and autophagy in drug resistance of AML patients, we investigated the correlation between autophagy‐related genes and CD34 marker. However, no significant association was observed between the expression of *MAP1LC3B*, *ATG5*, *ATG10*, *RB1CC1*, and *AMBRA1* genes and CD34 marker (*p* > 0.05) (Figure 5).

We recommend that future studies focus on the correlation of autophagy‐related genes with the prognosis of AML patients. We could not evaluate the levels of LC3, ATG5, ATG10, RB1CC1, and AMBRA1 proteins because of some limitations. Therefore, future studies need to measure these proteins using the western blot technique. Also, further studies are needed to investigate the relationship between autophagy‐related genes and minimal residual disease (MRD).

## 5 CONCLUSION

The present results showed that the key autophagy‐related genes, including *MAP1LC3B*, *ATG5*, *ATG10*, *RB1CC1*, and *AMBRA1*, could be related to the pathogenesis of AML and the patients' response to treatment. Therefore, these genes may be suggested as therapeutic biomarkers to follow‐up the CR status. Also, inhibition of autophagy‐related genes might be beneficial in inducing remission, preventing relapse, and also, improving the treatment outcomes of AML patients.

## CONFLICT OF INTEREST

The authors declare no conflict of interest.

## AUTHOR CONTRIBUTIONS

Parisa Tandel designed the study, performed the research, analyzed the data, and wrote the manuscript. Gholamhossein Tamaddon designed the study and was responsible for data management and analysis. Reza Ranjbaran revised the article critically and contributed to data analysis. Eqbal Ebrahimi performed the research and analyzed the data. Mani Ramzi and Alireza Rezvani revised the article critically.

## ETHICAL COMPLIANCE

The local Ethics Committee of Shiraz University of Medical Sciences approved this study (IR.SUMS.REC.1397.085), and the written informed consent was obtained from all participants.

## Data Availability

The datasets generated during and analyzed during the current study are available from the corresponding author on reasonable request.
